# Implementation Benchmark of Tumor-Agnostic Eligibility Signals Across Routine Comprehensive Genomic Profiling Platforms in Japan: A Nationwide C-CAT Analysis

**DOI:** 10.3390/curroncol33060324

**Published:** 2026-05-30

**Authors:** Shinya Kajiura, Naohiko Nakamura, Ryuji Hayashi

**Affiliations:** Department of Medical Oncology and Palliative Medicine, Toyama University Hospital, 2630 Sugitani, Toyama 930-0194, Toyama, Japan; naohiko@med.u-toyama.ac.jp (N.N.); hsayaka@med.u-toyama.ac.jp (R.H.)

**Keywords:** precision oncology, tumor-agnostic biomarker, comprehensive genomic profiling, C-CAT, Japan, real-world data, implementation benchmark, liquid biopsy

## Abstract

Routine precision oncology requires realistic benchmarks for tumor-agnostic eligibility signals observed in heterogeneous comprehensive genomic profiling (CGP) pathways. We performed a retrospective descriptive analysis of anonymized aggregated nationwide Center for Cancer Genomics and Advanced Therapeutics (C-CAT) data in Japan, including 97,343 CGP-tested cases summarized across five routine CGP platforms and categorized into 12 prespecified organ groups for analysis. The primary strict approved set endpoint was the case-level union of MSI-H, TMB-H, NTRK fusion/rearrangement, RET fusion/rearrangement, and ERBB2 amplification; the expanded practical set endpoint additionally included ALK fusion/rearrangement and BRAF V600E. The primary strict approved set endpoint was observed in 14,005 cases (14.4%), and the expanded practical set endpoint in 15,911 cases (16.3%), adding 1906 cases and increasing the observed rate by 2.0 percentage points. Signals varied across organ groups and platform/specimen contexts. TMB-H and ERBB2 amplification numerically dominated the primary set signal, whereas NTRK and RET fusion/rearrangement remained rare. These observed frequencies should be interpreted as case-level implementation signals surfaced through routine CGP rather than assay superiority evidence, biological prevalence estimates, or treatment-benefit data. This nationwide, platform-aware benchmark supports practical interpretation of tumor-agnostic eligibility signals in routine CGP practice in Japan.

## Simple Summary

Comprehensive genomic profiling (CGP) tests are now used in routine cancer care in Japan to look for molecular signals that may guide treatment discussions across cancer types. Clinicians and molecular tumor boards need realistic expectations about how often these signals are found in everyday testing. In this nationwide C-CAT analysis of 97,343 CGP-tested cases, at least one signal in the primary strict approved set was observed in 14.4% of cases. When two additional practice-oriented alterations were added, the expanded practical set rate was 16.3%. The observed frequencies differed by organ group, platform, and specimen/testing context, emphasizing the importance of interpreting CGP findings within the clinical testing pathway. This nationwide benchmark may support clinicians and molecular tumor boards in interpreting CGP reports and counseling patients in routine cancer genomic medicine.

## 1. Introduction

Precision oncology increasingly links treatment eligibility to molecular features that may be shared across tumor types, rather than to tissue of origin alone. Tumor-agnostic biomarkers such as MSI-H, TMB-H, and oncogenic fusions have therefore become important for clinical trial interpretation, regulatory decision-making, and routine genomic testing interpretation [1]. The clinical relevance of this framework is illustrated by durable activity of targeted agents in NTRK fusion-positive and RET fusion-positive cancers [2,3].

In Japan, CGP has been implemented as part of a national cancer genomic medicine framework linked to C-CAT, which aggregates clinical genomic information generated through routine care [4,5]. This setting differs from a single-protocol screening study: multiple tissue-based and liquid-based CGP platforms are used across diverse disease, specimen, and clinical pathway contexts [6,7]. Consequently, clinicians and molecular tumor boards need a benchmark that reflects what is actually surfaced through routine testing in this nationwide system.

Such a benchmark must be interpreted in light of platform and specimen context. Fusion ascertainment can depend on assay design, specimen type, and sequencing strategy, as highlighted in professional recommendations for NTRK and RET testing [8,9]. Similarly, tissue availability, tumor fraction, circulating tumor DNA shedding, panel architecture, analytic pipelines, and reporting rules can influence which signals are observed in routine practice [10,11]. These considerations make it inappropriate to treat platform-level differences as direct evidence of assay superiority, equivalent sensitivity, or biological prevalence.

Against this background, we performed a retrospective descriptive analysis of anonymized aggregated nationwide C-CAT data. The primary objective was to characterize the observed case-level frequency and landscape of a Japan-specific primary strict approved set endpoint across five routine CGP platforms and 12 prespecified organ groups used for this analysis. This primary endpoint was defined according to the Japan-specific regulatory context at the time the endpoint framework was prespecified and comprised MSI-H, TMB-H, NTRK fusion/rearrangement, RET fusion/rearrangement, and ERBB2 amplification. A secondary expanded practical set analysis added ALK fusion/rearrangement and BRAF V600E as adjacent practice-oriented alterations; subsequent Japanese tumor-agnostic approval for ALK fusion-positive solid tumors further supports the clinical relevance of retaining ALK in this secondary framework without changing the prespecified primary endpoint. The study was designed as a platform-aware, practice-facing benchmark for routine CGP interpretation, not as an assay performance comparison, a biological prevalence estimate, or a treatment effect study.

## 2. Materials and Methods

### 2.1. Study Design and Data Source

This retrospective descriptive study used anonymized aggregated clinico-genomic data from the Center for Cancer Genomics and Advanced Therapeutics (C-CAT), the national data center for cancer genomic medicine in Japan. C-CAT aggregates information generated through comprehensive genomic profiling (CGP) performed under the Japanese cancer genomic medicine system and therefore reflects routine nationwide clinical practice. The analytic dataset summarized CGP-tested cases available through 31 March 2025. The analysis was designed to describe observed case-level tumor-agnostic eligibility signals across routine CGP platforms and prespecified organ group categories in the Japanese cancer genomic medicine system.

### 2.2. Study Population and Organ Group Classification

The analytic unit was the registered CGP-tested case as represented in the nationwide aggregated dataset. Under the Japanese cancer genomic medicine system, CGP is generally reimbursed once per patient in routine practice, so registered CGP-tested cases are expected to largely correspond to unique patients within this framework. Cases were classified into 12 prespecified organ groups used for this analysis in the following order: biliary tract, bowel, breast, esophagogastric, gynecologic, head and neck/thyroid, liver, central/peripheral nervous system, other, pancreas, thoracic, and genitourinary. The analytic denominator consisted of all registered CGP-tested cases represented in the aggregated outputs available to the authors through the data cutoff. Denominators were defined separately for the overall cohort, each platform, each organ group, each organ-by-platform cell, and the pooled tissue-based and liquid-based testing context summaries.

### 2.3. Biomarker Definitions and Endpoint Framework

The primary strict approved set was prespecified as the primary endpoint and comprised microsatellite instability-high (MSI-H), tumor mutational burden-high (TMB-H), NTRK fusion/rearrangement, RET fusion/rearrangement, and ERBB2 amplification within a Japan-specific strict approved set framework. The expanded practical set was a prespecified secondary framework comprising the full primary set plus ALK fusion/rearrangement and BRAF V600E, included to capture adjacent actionable alterations that may influence real-world interpretation of CGP results. Union endpoints were used to avoid double counting when more than one biomarker class co-occurred in the same case. Biomarker classes were operationalized from the cumulative nationwide outputs as follows: MSI-H was counted when MSI-Status was reported as high or equivocal; TMB-H when TMB-Status was reported as high or, if blank, when the reported TMB value was at least 10 mutations/Mb; NTRK positivity was restricted to rearrangement class events annotated as fusion in NTRK1, NTRK2, or NTRK3; RET positivity to rearrangement class RET fusion/rearrangement events; ERBB2 positivity to copy number amplification; ALK positivity in the expanded framework to fusion/rearrangement events; and BRAF positivity in the expanded framework to BRAF V600E. Biomarker-specific frequencies were summarized separately because biomarker domains could overlap within the same case, whereas each union endpoint counted an eligible case once. Detailed operational definitions and interpretation notes are provided in Supplementary Table S4.

### 2.4. CGP Platform Classification

Platform names were standardized into five manuscript-level CGP categories in a fixed order: FoundationOne CDx, FoundationOne Liquid CDx, GenMineTOP, NCC Oncopanel, and Guardant360. For pooled specimen context summaries, platforms were additionally grouped as tissue-based CGP (FoundationOne CDx, GenMineTOP, and NCC Oncopanel) and liquid-based CGP (FoundationOne Liquid CDx and Guardant360). These classifications were applied consistently across overall, organ group, and organ-by-platform summaries. Because platform use in routine practice was non-random, platform-specific and pooled specimen context summaries were treated as descriptive testing context summaries. Platform and assay context considerations for interpreting these descriptive summaries are provided in Supplementary Table S5.

### 2.5. Statistical Analysis

Observed frequency was calculated as the number of positive cases divided by the total number of cases within each analytic category. Descriptive summaries were prepared overall, by CGP platform, by organ group, by organ–platform combination, and for pooled tissue-based versus liquid-based CGP. Exact binomial 95% confidence intervals (Clopper–Pearson) were calculated for the categories in Table 1. No hypothesis testing was performed for pooled tissue-based versus liquid-based summaries. Analyses were performed using R version 4.5.2 (R Foundation for Statistical Computing, Vienna, Austria). Organ-by-platform cell denominators were derived from the same cumulative aggregated output used for endpoint tabulation and are displayed as cell-level denominators in Supplementary Table S1. No regression modeling or formal comparative modeling was performed because the study was designed as an aggregated descriptive benchmark.

### 2.6. Ethics and Informed Consent

This study used anonymized data derived from the C-CAT framework. Under the Japanese cancer genomic medicine system, patients provide informed consent before registration in C-CAT. This secondary analysis used anonymized aggregated data obtained through the C-CAT data use framework and was conducted in accordance with the relevant governance framework for secondary use of C-CAT data in Japan. The data were accessed for research purposes on 29 January 2026, and the authors did not have access to information that could identify individual participants during or after data collection. The study was approved by the Institutional Review Board of Toyama University on 1 May 2024 (Approval No. R2024016) and the Information Utilization Review Board of C-CAT in August 2025 (Approval No. CDU2025-010N) and was conducted in accordance with the Declaration of Helsinki, as revised in 2013.

## 3. Results

### 3.1. Overall Cohort and Primary Any Positive Rate with Secondary Expanded Set Context

In the aggregated nationwide cohort of 97,343 CGP-tested cases, the primary strict approved set endpoint was observed in 14,005 cases (14.4%; 95% CI, 14.2–14.6%) (Table 1). This case-level union endpoint comprised MSI-H, TMB-H, NTRK fusion/rearrangement, RET fusion/rearrangement, and ERBB2 amplification. In the secondary expanded practical set endpoint, which additionally incorporated ALK fusion/rearrangement and BRAF V600E, the any positive count increased to 15,911 cases (16.3%; 95% CI, 16.1–16.6%), corresponding to 1906 additional cases and a 2.0-percentage-point increase. Because cases with more than one biomarker class were counted once in each union endpoint, these values summarize observed case-level eligibility signal frequencies rather than biomarker-specific totals.

### 3.2. Platform-Specific Observed Any Positive Rates

**Figure 1:**
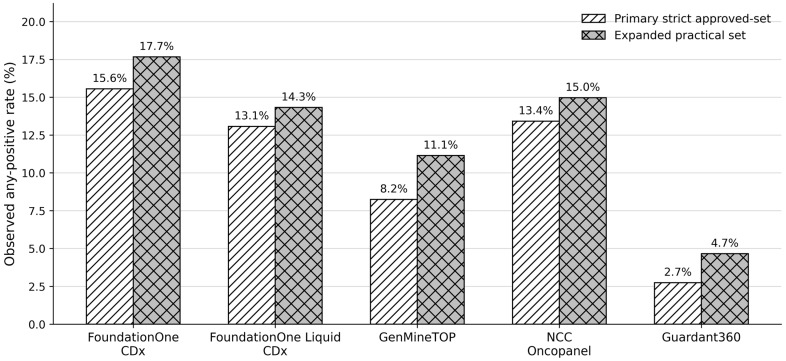
Platform-specific observed any positive rates for the primary strict approved set and expanded practical set endpoints. Paired bars show observed case-level frequencies for the five standardized CGP platforms in the fixed manuscript order; the expanded practical set endpoint adds ALK fusion/rearrangement and BRAF V600E to the primary strict approved set endpoint.

Platform-specific observed primary any positive rates were 15.6% for FoundationOne CDx (10,421/66,992), 13.1% for FoundationOne Liquid CDx (1945/14,878), 8.2% for GenMineTOP (349/4235), 13.4% for NCC Oncopanel (1234/9196), and 2.7% for Guardant360 (56/2042), in the fixed manuscript order. Corresponding expanded practical set rates were 17.7%, 14.3%, 11.1%, 15.0%, and 4.7%, respectively (Table 1; Figure 1). Pooled tissue-based and liquid-based summaries were 14.9% and 11.8% for the primary endpoint and 17.0% and 13.2% for the expanded endpoint, respectively.

### 3.3. Organ Group-Specific Patterns

**Figure 2:**
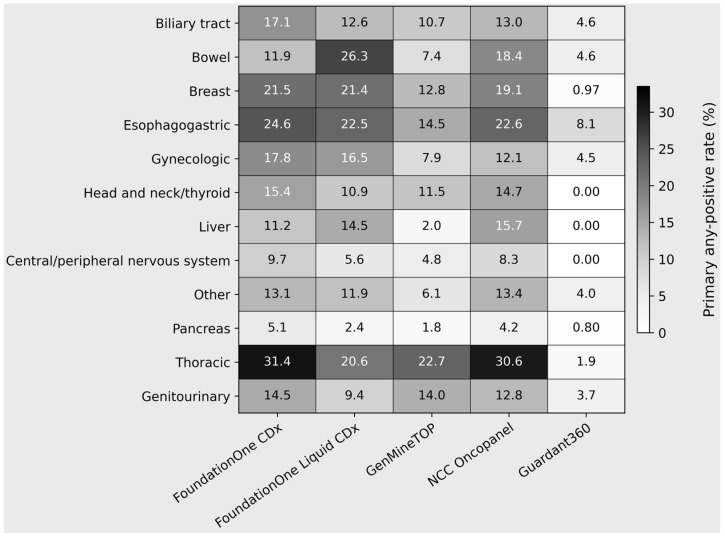
Organ-by-platform matrix of the primary strict approved set any positive rate. Annotated grayscale tiles show observed case-level frequencies for each organ group-by-platform cell; cell-level denominators and positive counts are provided in Supplementary Table S1. Cell-level denominators should be considered when interpreting low-frequency or zero-event cells.

Organ-level primary any positive rates ranged from 4.0% in pancreas to 27.7% in thoracic tumors, with other notable values including 23.6% in esophagogastric tumors and 20.8% in breast tumors (Table 2). In the fixed manuscript order, rates were 15.3% for biliary tract, 13.5% for bowel, 20.8% for breast, 23.6% for esophagogastric, 16.8% for gynecologic, 14.6% for head and neck/thyroid, 11.5% for liver, 8.6% for central/peripheral nervous system, 12.4% for other, 4.0% for pancreas, 27.7% for thoracic, and 12.7% for genitourinary tumors. The organ-by-platform heatmap further illustrates within-organ variation across testing contexts (Figure 2). Cell-level denominators and primary positive counts for the heatmap cells are provided in Supplementary Table S1. The organ-specific distribution of platform use is shown in Supplementary Figure S2 as context for the organ-by-platform display.

The expanded practical set endpoint produced limited changes in most organ groups but larger uplifts in head and neck/thyroid tumors (+469 cases; +11.6 pp), bowel tumors (+690 cases; +4.4 pp), and central/peripheral nervous system tumors (+171 cases; +5.1 pp) (Table 2; Supplementary Figure S1). Added percentage points in the remaining organ groups ranged from 0.2 to 2.3 pp.

### 3.4. Biomarker Domain Context for the Observed Landscape

**Figure 3:**
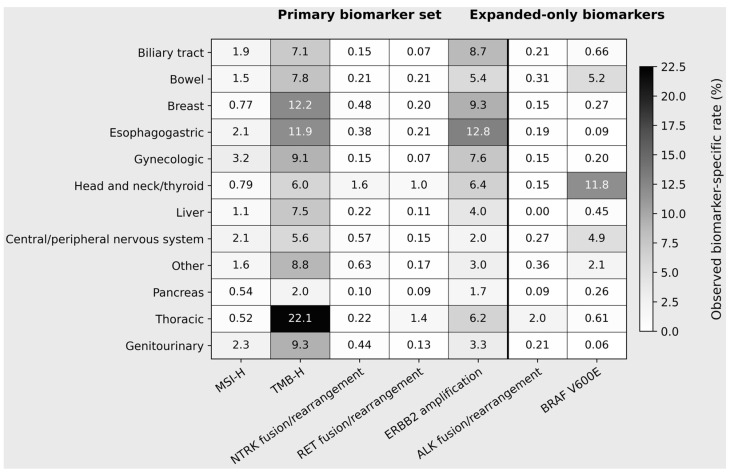
Biomarker-specific organ-level observed frequency matrix. Grayscale tiles show observed frequencies for the five primary strict approved set biomarkers and the two expanded practical set biomarkers. ALK fusion/rearrangement and BRAF V600E are expanded set biomarkers only. Overlap among biomarker domains is summarized in Supplementary Table S3.

TMB-H and ERBB2 amplification accounted for the largest biomarker-specific frequencies within the primary set (8.61% and 5.67% of the cohort, respectively), whereas NTRK fusion/rearrangement and RET fusion/rearrangement were rare (0.34% and 0.26%) (Figure 3; Supplementary Table S2). ERBB2 amplification was prominent in several gastrointestinal and breast-related strata, and TMB-H showed its highest organ-level frequency in thoracic tumors. Among the added expanded set biomarkers, BRAF V600E was more frequent than ALK fusion/rearrangement (1.91% vs. 0.33%), aligning with the larger expanded set uplifts in bowel and head and neck/thyroid tumors.

### 3.5. Overlap and Supplementary Descriptive Context

Overlap across biomarker classes was observed in both endpoint definitions: 1968 primary set cases and 2168 expanded set cases had two or more positive biomarker classes (Supplementary Table S3). Consequently, biomarker-specific counts exceeded the corresponding any positive union endpoints and are not additive components of those endpoints. The cumulative outputs supported counts by number of positive biomarker classes but not exact combination-level overlap matrices.

## 4. Discussion

In this nationwide aggregated analysis of 97,343 CGP-tested cases within the Japanese C-CAT framework, the primary strict approved set endpoint was observed in 14.4% of cases, and the secondary expanded practical set endpoint was observed in 16.3%, adding 1906 cases and 2.0 percentage points. Observed frequencies varied across organ groups and platform/specimen contexts. TMB-H and ERBB2 amplification numerically accounted for much of the primary set signal, whereas NTRK and RET fusion/rearrangement were uncommon. The main contribution of this study is to provide a nationwide, platform-aware benchmark for how tumor-agnostic eligibility signals are surfaced through routine cancer genomic medicine in Japan [1,4,5].

For clinicians and molecular tumor boards, this benchmark is useful for expectation-setting. It helps place an individual CGP result within the broader national testing landscape, showing which organ and testing contexts more often yielded a strict approved set signal and where lower observed yields were common. In this way, the benchmark can support more nuanced counseling and molecular tumor board discussion for both positive and negative CGP reports.

The primary strict approved set endpoint was anchored to the Japan-specific regulatory context at the time the endpoint framework was prespecified and at the time of original submission. This primary set comprised MSI-H, TMB-H, NTRK fusion/rearrangement, RET fusion/rearrangement, and ERBB2 amplification. The expanded practical set was intentionally kept separate and added ALK fusion/rearrangement and BRAF V600E as practice-oriented alterations that may affect real-world interpretation of CGP reports. The subsequent Japanese tumor-agnostic approval for ALK fusion-positive solid tumors further supports the clinical relevance of including ALK in this secondary framework, while preserving the stability of the prespecified primary endpoint. BRAF V600E was similarly retained in the expanded practical set as a clinically familiar actionable alteration discussed across multiple tumor contexts.

The organ-specific patterns are clinically informative. Higher observed primary rates in thoracic, esophagogastric, and breast groups are consistent with the contributions of TMB-H and ERBB2 amplification, whereas the lower pancreatic signal illustrates that common clinical testing contexts may still yield fewer strict approved set findings. Rare NTRK and RET signals contributed little to the overall percentage, but their detection remains important because a single fusion/rearrangement can open a highly relevant treatment discussion [1,5,12].

Platform and specimen context are also part of the implementation signal. Differences across platform-specific summaries may reflect a combination of assay scope, analyte type, specimen availability, tumor fraction, reporting rules, organ mix, and clinical pathway. These differences do not make the platform-aware tabulations uninformative; rather, they show why observed CGP signals should be interpreted in the context of how testing is deployed in daily practice. The prior literature on liquid biopsy implementation and ctDNA detection underscores these context-dependent effects [13,14]. C-CAT and Japanese real-world CGP reports further support the need for platform-aware interpretation in the national implementation setting [7,10].

This study has several strengths. It leverages a large nationwide routine-practice C-CAT dataset, includes five standardized CGP platforms and 12 prespecified organ groups used for this analysis, and evaluates a prespecified primary strict approved set endpoint alongside a clearly separated expanded practical set endpoint. The case-level union endpoint is clinically intuitive because it asks whether at least one relevant eligibility signal was surfaced for a tested case and avoids double counting when multiple biomarker classes coexist [5,15,16].

Several limitations define the proper use of this benchmark. The study was retrospective and based on anonymized aggregated data; patient-level linkage, clinical covariate adjustment, treatment selection, treatment response, survival outcomes, prospective validation, and paired tissue/plasma or cross-platform concordance assessment were not available. Platform use was non-random, so observed platform/specimen differences cannot be disentangled from specimen context, organ mix, testing pathway, or other selection factors. Broad organ groups may smooth biologically distinct tumor types. Biomarker classes were operationalized from cumulative outputs, including MSI-H counting of high or equivocal MSI status and TMB-H counting based on available TMB status/value fields; therefore, biomarker evaluability and reporting behavior were not fully symmetric across platforms. An exact combination-level overlap matrix was also unavailable. These limitations mean that the findings should be used as a routine CGP implementation benchmark rather than as biological prevalence estimates, assay performance rankings, or treatment-effect evidence.

In conclusion, this study provides a nationwide, platform-aware benchmark for tumor-agnostic eligibility signals observed through routine CGP in Japan. The primary strict approved set endpoint offers a stable Japan-specific benchmark, and the expanded practical set analysis adds useful secondary context for adjacent actionable alterations without redefining the primary endpoint. By showing how these signals are surfaced across organ groups, platforms, and specimen/testing contexts, the findings support expectation-setting for clinicians, molecular tumor boards, and genomic medicine planning, and provide a practical reference point for the continuing development of cancer genomic medicine in Japan [4,5].

## Figures and Tables

**Table 1:** Overall, platform-specific, and pooled specimen context observed frequencies of any tumor-agnostic biomarker positivity.

Group	Cases	Primary *n*/*N* (%)	Primary 95% CI	Expanded *n*/*N* (%)	Expanded 95% CI	Added Cases	Added pp
Overall cohort	97,343	14,005/97,343 (14.4%)	14.2–14.6%	15,911/97,343 (16.3%)	16.1–16.6%	1906	2.0 pp
FoundationOne CDx	66,992	10,421/66,992 (15.6%)	15.3–15.8%	11,837/66,992 (17.7%)	17.4–18.0%	1416	2.1 pp
FoundationOne Liquid CDx	14,878	1945/14,878 (13.1%)	12.5–13.6%	2131/14,878 (14.3%)	13.8–14.9%	186	1.3 pp
GenMineTOP	4235	349/4235 (8.2%)	7.4–9.1%	472/4235 (11.1%)	10.2–12.1%	123	2.9 pp
NCC Oncopanel	9196	1234/9196 (13.4%)	12.7–14.1%	1376/9196 (15.0%)	14.2–15.7%	142	1.5 pp
Guardant360	2042	56/2042 (2.7%)	2.1–3.5%	95/2042 (4.7%)	3.8–5.7%	39	1.9 pp
Liquid-based pooled	16,920	2001/16,920 (11.8%)	11.3–12.3%	2226/16,920 (13.2%)	12.7–13.7%	225	1.3 pp
Tissue-based pooled	80,423	12,004/80,423 (14.9%)	14.7–15.2%	13,685/80,423 (17.0%)	16.8–17.3%	1681	2.1 pp

Primary strict approved set endpoint = case-level union of MSI-H, TMB-H, NTRK fusion/rearrangement, RET fusion/rearrangement, and ERBB2 amplification. Expanded practical set endpoint = the primary set plus ALK fusion/rearrangement and BRAF V600E. Percentages are observed case-level frequencies; confidence intervals are exact binomial 95% confidence intervals. Pooled tissue-based and liquid-based rows summarize testing context categories.

**Table 2:** Organ group-specific observed frequencies of any tumor-agnostic biomarker positivity.

Organ Group	Cases	Liquid Share	Primary *n*/*N* (%)	Expanded *n*/*N* (%)	Added Cases	Added pp
Biliary tract	9103	21.8%	1394/9103 (15.3%)	1464/9103 (16.1%)	70	0.8 pp
Bowel	15,791	12.0%	2130/15,791 (13.5%)	2820/15,791 (17.9%)	690	4.4 pp
Breast	7498	19.4%	1562/7498 (20.8%)	1586/7498 (21.2%)	24	0.3 pp
Esophagogastric	5823	12.9%	1372/5823 (23.6%)	1384/5823 (23.8%)	12	0.2 pp
Gynecologic	10,963	6.3%	1846/10,963 (16.8%)	1881/10,963 (17.2%)	35	0.3 pp
Head and neck/thyroid	4030	7.8%	590/4030 (14.6%)	1059/4030 (26.3%)	469	11.6 pp
Liver	897	17.8%	103/897 (11.5%)	107/897 (11.9%)	4	0.4 pp
Central/peripheral nervous system	3357	2.8%	288/3357 (8.6%)	459/3357 (13.7%)	171	5.1 pp
Other	9369	7.7%	1158/9369 (12.4%)	1367/9369 (14.6%)	209	2.2 pp
Pancreas	15,270	29.2%	616/15,270 (4.0%)	664/15,270 (4.3%)	48	0.3 pp
Thoracic	6740	25.6%	1868/6740 (27.7%)	2022/6740 (30.0%)	154	2.3 pp
Genitourinary	8502	31.5%	1078/8502 (12.7%)	1098/8502 (12.9%)	20	0.2 pp

Primary and expanded endpoints are defined as in Table 1. Liquid share is the proportion of testing performed on pooled liquid-based platforms within each organ group and is provided as descriptive context.

## Data Availability

The authors do not control the underlying C-CAT database. The data used in this study were obtained as anonymized aggregated counts through the approved C-CAT data use framework and are subject to institutional and data provider governance restrictions. Therefore, the underlying source-level C-CAT data are not publicly available from the authors. Access to source-level C-CAT data requires formal application to C-CAT and the relevant institutional approvals under the Japanese cancer genomic medicine governance framework.

## Supplementary Materials

The following supporting information can be downloaded at: https://www.mdpi.com/article/10.3390/curroncol33060324/s1, Table S1: Complete organ-group × platform matrix for the primary strict approved-set and expanded practical-set any-positive endpoints; Table S2: Biomarker-specific observed frequencies by organ group and platform; Table S3: Distribution of the number of positive biomarkers per case overall and by organ group; Table S4: Operational definitions of biomarker domains and case-level union endpoints; Table S5: Platform and assay-context considerations for interpreting biomarker assessment across routine CGP platforms; Figure S1: Organ-by-platform matrix of the expanded practical-set any-positive rate; Figure S2: Platform-use distribution across organ groups.
