# Supplementary material for: Implementation Benchmark of Tumor-Agnostic Eligibility Signals Across Routine Comprehensive Genomic Profiling Platforms in Japan: A Nationwide C-CAT Analysis
Source: Curr Oncol. 2026 May 30;33(6):324. doi: 10.3390/curroncol33060324 (PMC13297875; doi:10.3390/curroncol33060324)
Supplement: Supplementary file 1 [file curroncol-33-00324-s001.zip › 33CO_SupplFigureLegends20260418.pdf]

Supplementary Figure Legends

Supplementary Figure S1. Organ-by-platform matrix of the expanded any-positive rate.

This matrix parallels Figure 2 but maps the expanded practical-set any-positive rate after adding ALK fusion/rearrangement and BRAF V600E to the strict approved-set primary biomarker universe. Annotated grayscale tiles are used to preserve legibility in black-and-white print.

Supplementary Figure S2. Platform-use distribution across organ groups.

Each horizontal stacked bar shows the organ-specific distribution of testing volume across the five standardized CGP platforms. The figure is provided to contextualize organ-by-platform heterogeneity in the observed positivity matrices; it does not imply random platform allocation or comparability of tested populations.
