# Supplementary material for: Implementation Benchmark of Tumor-Agnostic Eligibility Signals Across Routine Comprehensive Genomic Profiling Platforms in Japan: A Nationwide C-CAT Analysis
Source: Curr Oncol. 2026 May 30;33(6):324. doi: 10.3390/curroncol33060324 (PMC13297875; doi:10.3390/curroncol33060324)
Supplement: Supplementary file 1 [file curroncol-33-00324-s001.zip › 33CO_SupplTables20260419a.pdf]

Supplementary Tables

Supplementary Table S1. Complete organ-group × platform matrix for the primary strict approved-set and expanded practical-set any-positive endpoints

Organ group	Platform	Specimen context	Cases	Primary n/N (%)	Expanded n/N (%)	Added cases	Added pp
Biliary tract	FoundationOne CDx	Tissue Based	5884	1,009/5,884 (17.1%)	1,059/5,884 (18.0%)	50	0.8 pp
Biliary tract	FoundationOne Liquid CDx	Liquid Based	1727	218/1,727 (12.6%)	227/1,727 (13.1%)	9	0.5 pp
Biliary tract	GenMineTOP	Tissue Based	243	26/243 (10.7%)	29/243 (11.9%)	3	1.2 pp
Biliary tract	NCC Oncopanel	Tissue Based	990	129/990 (13.0%)	136/990 (13.7%)	7	0.7 pp
Biliary tract	Guardant360	Liquid Based	259	12/259 (4.6%)	13/259 (5.0%)	1	0.4 pp
Bowel	FoundationOne CDx	Tissue Based	12000	1,426/12,000 (11.9%)	1,946/12,000 (16.2%)	520	4.3 pp
Bowel	FoundationOne Liquid CDx	Liquid Based	1543	406/1,543 (26.3%)	477/1,543 (30.9%)	71	4.6 pp
Bowel	GenMineTOP	Tissue Based	610	45/610 (7.4%)	75/610 (12.3%)	30	4.9 pp
Bowel	NCC Oncopanel	Tissue Based	1288	237/1,288 (18.4%)	282/1,288 (21.9%)	45	3.5 pp
Bowel	Guardant360	Liquid Based	350	16/350 (4.6%)	40/350 (11.4%)	24	6.9 pp
Breast	FoundationOne CDx	Tissue Based	5311	1,142/5,311 (21.5%)	1,155/5,311 (21.7%)	13	0.2 pp
Breast	FoundationOne Liquid CDx	Liquid Based	1349	289/1,349 (21.4%)	300/1,349 (22.2%)	11	0.8 pp

Organ group	Platform	Specimen context	Cases	Primary n/N (%)	Expanded n/N (%)	Added cases	Added pp
Breast	GenMineTOP	Tissue Based	164	21/164 (12.8%)	21/164 (12.8%)	0	0.0 pp
Breast	NCC Oncopanel	Tissue Based	571	109/571 (19.1%)	109/571 (19.1%)	0	0.0 pp
Breast	Guardant360	Liquid Based	103	1/103 (1.0%)	1/103 (1.0%)	0	0.0 pp
Esophagogastric	FoundationOne CDx	Tissue Based	4259	1,048/4,259 (24.6%)	1,056/4,259 (24.8%)	8	0.2 pp
Esophagogastric	FoundationOne Liquid CDx	Liquid Based	654	147/654 (22.5%)	149/654 (22.8%)	2	0.3 pp
Esophagogastric	GenMineTOP	Tissue Based	173	25/173 (14.5%)	26/173 (15.0%)	1	0.6 pp
Esophagogastric	NCC Oncopanel	Tissue Based	638	144/638 (22.6%)	145/638 (22.7%)	1	0.2 pp
Esophagogastric	Guardant360	Liquid Based	99	8/99 (8.1%)	8/99 (8.1%)	0	0.0 pp
Gynecologic	FoundationOne CDx	Tissue Based	9008	1,607/9,008 (17.8%)	1,636/9,008 (18.2%)	29	0.3 pp
Gynecologic	FoundationOne Liquid CDx	Liquid Based	643	106/643 (16.5%)	109/643 (17.0%)	3	0.5 pp
Gynecologic	GenMineTOP	Tissue Based	534	42/534 (7.9%)	43/534 (8.1%)	1	0.2 pp
Gynecologic	NCC Oncopanel	Tissue Based	734	89/734 (12.1%)	91/734 (12.4%)	2	0.3 pp
Gynecologic	Guardant360	Liquid Based	44	2/44 (4.5%)	2/44 (4.5%)	0	0.0 pp
Head and neck/thyroid	FoundationOne CDx	Tissue Based	3159	485/3,159 (15.4%)	882/3,159 (27.9%)	397	12.6 pp
Head and neck/thyroid	FoundationOne Liquid CDx	Liquid Based	294	32/294 (10.9%)	48/294 (16.3%)	16	5.4 pp

Organ group	Platform	Specimen context	Cases	Primary n/N (%)	Expanded n/N (%)	Added cases	Added pp
Head and neck/thyroid	GenMineTOP	Tissue Based	262	30/262 (11.5%)	52/262 (19.8%)	22	8.4 pp
Head and neck/thyroid	NCC Oncopanel	Tissue Based	293	43/293 (14.7%)	75/293 (25.6%)	32	10.9 pp
Head and neck/thyroid	Guardant360	Liquid Based	22	0/22 (0.0%)	2/22 (9.1%)	2	9.1 pp
Liver	FoundationOne CDx	Tissue Based	605	68/605 (11.2%)	70/605 (11.6%)	2	0.3 pp
Liver	FoundationOne Liquid CDx	Liquid Based	145	21/145 (14.5%)	23/145 (15.9%)	2	1.4 pp
Liver	GenMineTOP	Tissue Based	49	1/49 (2.0%)	1/49 (2.0%)	0	0.0 pp
Liver	NCC Oncopanel	Tissue Based	83	13/83 (15.7%)	13/83 (15.7%)	0	0.0 pp
Liver	Guardant360	Liquid Based	15	0/15 (0.0%)	0/15 (0.0%)	0	0.0 pp
Central/peripheral nervous system	FoundationOne CDx	Tissue Based	2425	235/2,425 (9.7%)	347/2,425 (14.3%)	112	4.6 pp
Central/peripheral nervous system	FoundationOne Liquid CDx	Liquid Based	90	5/90 (5.6%)	5/90 (5.6%)	0	0.0 pp
Central/peripheral nervous system	GenMineTOP	Tissue Based	608	29/608 (4.8%)	68/608 (11.2%)	39	6.4 pp
Central/peripheral nervous system	NCC Oncopanel	Tissue Based	229	19/229 (8.3%)	39/229 (17.0%)	20	8.7 pp
Central/peripheral nervous system	Guardant360	Liquid Based	5	0/5 (0.0%)	0/5 (0.0%)	0	0.0 pp
Other	FoundationOne CDx	Tissue Based	6935	910/6,935 (13.1%)	1,071/6,935 (15.4%)	161	2.3 pp
Other	FoundationOne Liquid CDx	Liquid Based	670	80/670 (11.9%)	96/670 (14.3%)	16	2.4 pp

Organ group	Platform	Specimen context	Cases	Primary n/N (%)	Expanded n/N (%)	Added cases	Added pp
Other	GenMineTOP	Tissue Based	881	54/881 (6.1%)	71/881 (8.1%)	17	1.9 pp
Other	NCC Oncopanel	Tissue Based	833	112/833 (13.4%)	127/833 (15.2%)	15	1.8 pp
Other	Guardant360	Liquid Based	50	2/50 (4.0%)	2/50 (4.0%)	0	0.0 pp
Pancreas	FoundationOne CDx	Tissue Based	8021	413/8,021 (5.1%)	435/8,021 (5.4%)	22	0.3 pp
Pancreas	FoundationOne Liquid CDx	Liquid Based	3707	89/3,707 (2.4%)	102/3,707 (2.8%)	13	0.4 pp
Pancreas	GenMineTOP	Tissue Based	340	6/340 (1.8%)	8/340 (2.4%)	2	0.6 pp
Pancreas	NCC Oncopanel	Tissue Based	2450	102/2,450 (4.2%)	110/2,450 (4.5%)	8	0.3 pp
Pancreas	Guardant360	Liquid Based	752	6/752 (0.8%)	9/752 (1.2%)	3	0.4 pp
Thoracic	FoundationOne CDx	Tissue Based	4260	1,336/4,260 (31.4%)	1,430/4,260 (33.6%)	94	2.2 pp
Thoracic	FoundationOne Liquid CDx	Liquid Based	1516	313/1,516 (20.6%)	347/1,516 (22.9%)	34	2.2 pp
Thoracic	GenMineTOP	Tissue Based	207	47/207 (22.7%)	52/207 (25.1%)	5	2.4 pp
Thoracic	NCC Oncopanel	Tissue Based	549	168/549 (30.6%)	180/549 (32.8%)	12	2.2 pp
Thoracic	Guardant360	Liquid Based	208	4/208 (1.9%)	13/208 (6.2%)	9	4.3 pp
Genitourinary	FoundationOne CDx	Tissue Based	5125	742/5,125 (14.5%)	750/5,125 (14.6%)	8	0.2 pp
Genitourinary	FoundationOne Liquid CDx	Liquid Based	2540	239/2,540 (9.4%)	248/2,540 (9.8%)	9	0.4 pp

Organ group	Platform	Specimen context	Cases	Primary n/N (%)	Expanded n/N (%)	Added cases	Added pp
Genitourinary	GenMineTOP	Tissue Based	164	23/164 (14.0%)	26/164 (15.9%)	3	1.8 pp
Genitourinary	NCC Oncopanel	Tissue Based	538	69/538 (12.8%)	69/538 (12.8%)	0	0.0 pp
Genitourinary	Guardant360	Liquid Based	135	5/135 (3.7%)	5/135 (3.7%)	0	0.0 pp

Values are extracted from the cumulative summaries for each organ-platform cell. Added percentage points refer to the absolute increase from the primary to the expanded endpoint within the same denominator. Cell-level denominators are provided in the Cases column and should be interpreted descriptively rather than as inferential sparse-cell thresholds.

Supplementary Table S2. Biomarker-specific observed frequencies by organ group and platform

Organ group	Platform	Cases	MSI-H	TMB-H	NTRK fusion/rearrangement	RET fusion/rearrangement	ERBB2 amplification	ALK fusion/rearrangement	BRAF V600E
Biliary tract	All Platforms	9103	176 (1.9%)	642 (7.1%)	14 (0.15%)	6 (0.07%)	791 (8.7%)	19 (0.21%)	60 (0.66%)
Biliary tract	FoundationOne CDx	5884	129 (2.2%)	378 (6.4%)	11 (0.19%)	5 (0.08%)	660 (11.2%)	11 (0.19%)	42 (0.71%)
Biliary tract	FoundationOne Liquid CDx	1727	26 (1.5%)	152 (8.8%)	3 (0.17%)	1 (0.06%)	76 (4.4%)	7 (0.41%)	7 (0.41%)
Biliary tract	GenMineTOP	243	0 (0.00%)	16 (6.6%)	0 (0.00%)	0 (0.00%)	10 (4.1%)	0 (0.00%)	3 (1.2%)
Biliary tract	NCC Oncopanel	990	18 (1.8%)	96 (9.7%)	0 (0.00%)	0 (0.00%)	36 (3.6%)	1 (0.10%)	7 (0.71%)

Organ group	Platform	Cases	MSI-H	TMB-H	NTRK fusion/rearrangement	RET fusion/rearrangement	ERBB2 amplification	ALK fusion/rearrangement	BRAF V600E
Biliary tract	Guardant360	259	3 (1.2%)	0 (0.00%)	0 (0.00%)	0 (0.00%)	9 (3.5%)	0 (0.00%)	1 (0.39%)
Bowel	All Platforms	15791	238 (1.5%)	1,225 (7.8%)	33 (0.21%)	33 (0.21%)	852 (5.4%)	49 (0.31%)	826 (5.2%)
Bowel	FoundationOne CDx	12000	200 (1.7%)	632 (5.3%)	21 (0.18%)	23 (0.19%)	742 (6.2%)	23 (0.19%)	598 (5.0%)
Bowel	FoundationOne Liquid CDx	1543	9 (0.58%)	365 (23.7%)	10 (0.65%)	8 (0.52%)	45 (2.9%)	23 (1.5%)	103 (6.7%)
Bowel	GenMineTOP	610	0 (0.00%)	26 (4.3%)	1 (0.16%)	0 (0.00%)	19 (3.1%)	1 (0.16%)	32 (5.2%)
Bowel	NCC Oncopanel	1288	24 (1.9%)	202 (15.7%)	1 (0.08%)	1 (0.08%)	36 (2.8%)	2 (0.16%)	65 (5.0%)
Bowel	Guardant360	350	5 (1.4%)	0 (0.00%)	0 (0.00%)	1 (0.29%)	10 (2.9%)	0 (0.00%)	28 (8.0%)
Breast	All Platforms	7498	58 (0.77%)	914 (12.2%)	36 (0.48%)	15 (0.20%)	699 (9.3%)	11 (0.15%)	20 (0.27%)
Breast	FoundationOne CDx	5311	48 (0.90%)	559 (10.5%)	25 (0.47%)	12 (0.23%)	623 (11.7%)	6 (0.11%)	9 (0.17%)
Breast	FoundationOne Liquid CDx	1349	10 (0.74%)	262 (19.4%)	9 (0.67%)	3 (0.22%)	31 (2.3%)	5 (0.37%)	11 (0.82%)
Breast	GenMineTOP	164	0 (0.00%)	12 (7.3%)	1 (0.61%)	0 (0.00%)	9 (5.5%)	0 (0.00%)	0 (0.00%)
Breast	NCC Oncopanel	571	0 (0.00%)	81 (14.2%)	1 (0.18%)	0 (0.00%)	35 (6.1%)	0 (0.00%)	0 (0.00%)
Breast	Guardant360	103	0 (0.00%)	0 (0.00%)	0 (0.00%)	0 (0.00%)	1 (0.97%)	0 (0.00%)	0 (0.00%)
Esophagogastric	All Platforms	5823	122 (2.1%)	695 (11.9%)	22 (0.38%)	12 (0.21%)	748 (12.8%)	11 (0.19%)	5 (0.09%)

Organ group	Platform	Cases	MSI-H	TMB-H	NTRK fusion/rearrangement	RET fusion/rearrangement	ERBB2 amplification	ALK fusion/rearrangement	BRAF V600E
Esophagogastric	FoundationOne CDx	4259	101 (2.4%)	452 (10.6%)	20 (0.47%)	10 (0.23%)	644 (15.1%)	8 (0.19%)	3 (0.07%)
Esophagogastric	FoundationOne Liquid CDx	654	6 (0.92%)	124 (19.0%)	2 (0.31%)	2 (0.31%)	41 (6.3%)	2 (0.31%)	1 (0.15%)
Esophagogastric	GenMineTOP	173	0 (0.00%)	11 (6.4%)	0 (0.00%)	0 (0.00%)	14 (8.1%)	1 (0.58%)	0 (0.00%)
Esophagogastric	NCC Oncopanel	638	12 (1.9%)	108 (16.9%)	0 (0.00%)	0 (0.00%)	44 (6.9%)	0 (0.00%)	1 (0.16%)
Esophagogastric	Guardant360	99	3 (3.0%)	0 (0.00%)	0 (0.00%)	0 (0.00%)	5 (5.1%)	0 (0.00%)	0 (0.00%)
Gynecologic	All Platforms	10963	348 (3.2%)	996 (9.1%)	16 (0.15%)	8 (0.07%)	828 (7.6%)	16 (0.15%)	22 (0.20%)
Gynecologic	FoundationOne CDx	9008	324 (3.6%)	793 (8.8%)	14 (0.16%)	7 (0.08%)	792 (8.8%)	13 (0.14%)	19 (0.21%)
Gynecologic	FoundationOne Liquid CDx	643	11 (1.7%)	99 (15.4%)	1 (0.16%)	1 (0.16%)	9 (1.4%)	3 (0.47%)	0 (0.00%)
Gynecologic	GenMineTOP	534	0 (0.00%)	30 (5.6%)	1 (0.19%)	0 (0.00%)	11 (2.1%)	0 (0.00%)	1 (0.19%)
Gynecologic	NCC Oncopanel	734	11 (1.5%)	74 (10.1%)	0 (0.00%)	0 (0.00%)	16 (2.2%)	0 (0.00%)	2 (0.27%)
Gynecologic	Guardant360	44	2 (4.5%)	0 (0.00%)	0 (0.00%)	0 (0.00%)	0 (0.00%)	0 (0.00%)	0 (0.00%)
Head and neck/thyroid	All Platforms	4030	32 (0.79%)	243 (6.0%)	65 (1.6%)	42 (1.0%)	258 (6.4%)	6 (0.15%)	476 (11.8%)
Head and neck/thyroid	FoundationOne CDx	3159	31 (0.98%)	186 (5.9%)	43 (1.4%)	34 (1.1%)	237 (7.5%)	6 (0.19%)	403 (12.8%)
Head and neck/thyroid	FoundationOne Liquid CDx	294	0 (0.00%)	21 (7.1%)	3 (1.0%)	4 (1.4%)	5 (1.7%)	0 (0.00%)	16 (5.4%)

Organ group	Platform	Cases	MSI-H	TMB-H	NTRK fusion/rearrangement	RET fusion/rearrangement	ERBB2 amplification	ALK fusion/rearrangement	BRAF V600E
Head and neck/thyroid	GenMineTOP	262	0 (0.00%)	9 (3.4%)	10 (3.8%)	2 (0.76%)	10 (3.8%)	0 (0.00%)	22 (8.4%)
Head and neck/thyroid	NCC Oncopanel	293	1 (0.34%)	27 (9.2%)	9 (3.1%)	2 (0.68%)	6 (2.0%)	0 (0.00%)	33 (11.3%)
Head and neck/thyroid	Guardant360	22	0 (0.00%)	0 (0.00%)	0 (0.00%)	0 (0.00%)	0 (0.00%)	0 (0.00%)	2 (9.1%)
Liver	All Platforms	897	10 (1.1%)	67 (7.5%)	2 (0.22%)	1 (0.11%)	36 (4.0%)	0 (0.00%)	4 (0.45%)
Liver	FoundationOne CDx	605	9 (1.5%)	36 (6.0%)	1 (0.17%)	0 (0.00%)	33 (5.5%)	0 (0.00%)	2 (0.33%)
Liver	FoundationOne Liquid CDx	145	1 (0.69%)	18 (12.4%)	0 (0.00%)	1 (0.69%)	3 (2.1%)	0 (0.00%)	2 (1.4%)
Liver	GenMineTOP	49	0 (0.00%)	1 (2.0%)	0 (0.00%)	0 (0.00%)	0 (0.00%)	0 (0.00%)	0 (0.00%)
Liver	NCC Oncopanel	83	0 (0.00%)	12 (14.5%)	1 (1.2%)	0 (0.00%)	0 (0.00%)	0 (0.00%)	0 (0.00%)
Liver	Guardant360	15	0 (0.00%)	0 (0.00%)	0 (0.00%)	0 (0.00%)	0 (0.00%)	0 (0.00%)	0 (0.00%)
Central/peripheral nervous system	All Platforms	3357	71 (2.1%)	189 (5.6%)	19 (0.57%)	5 (0.15%)	68 (2.0%)	9 (0.27%)	166 (4.9%)
Central/peripheral nervous system	FoundationOne CDx	2425	69 (2.8%)	148 (6.1%)	10 (0.41%)	3 (0.12%)	66 (2.7%)	9 (0.37%)	106 (4.4%)
Central/peripheral nervous system	FoundationOne Liquid CDx	90	0 (0.00%)	4 (4.4%)	0 (0.00%)	0 (0.00%)	2 (2.2%)	0 (0.00%)	0 (0.00%)
Central/peripheral nervous system	GenMineTOP	608	0 (0.00%)	19 (3.1%)	9 (1.5%)	1 (0.16%)	0 (0.00%)	0 (0.00%)	39 (6.4%)
Central/peripheral nervous system	NCC Oncopanel	229	2 (0.87%)	18 (7.9%)	0 (0.00%)	1 (0.44%)	0 (0.00%)	0 (0.00%)	21 (9.2%)

Organ group	Platform	Cases	MSI-H	TMB-H	NTRK fusion/rearrangement	RET fusion/rearrangement	ERBB2 amplification	ALK fusion/rearrangement	BRAF V600E
Central/peripheral nervous system	Guardant360	5	0 (0.00%)	0 (0.00%)	0 (0.00%)	0 (0.00%)	0 (0.00%)	0 (0.00%)	0 (0.00%)
Other	All Platforms	9369	149 (1.6%)	824 (8.8%)	59 (0.63%)	16 (0.17%)	277 (3.0%)	34 (0.36%)	196 (2.1%)
Other	FoundationOne CDx	6935	133 (1.9%)	614 (8.9%)	41 (0.59%)	14 (0.20%)	254 (3.7%)	28 (0.40%)	154 (2.2%)
Other	FoundationOne Liquid CDx	670	10 (1.5%)	68 (10.1%)	5 (0.75%)	0 (0.00%)	9 (1.3%)	1 (0.15%)	15 (2.2%)
Other	GenMineTOP	881	0 (0.00%)	39 (4.4%)	7 (0.79%)	2 (0.23%)	7 (0.79%)	4 (0.45%)	13 (1.5%)
Other	NCC Oncopanel	833	6 (0.72%)	103 (12.4%)	6 (0.72%)	0 (0.00%)	5 (0.60%)	1 (0.12%)	14 (1.7%)
Other	Guardant360	50	0 (0.00%)	0 (0.00%)	0 (0.00%)	0 (0.00%)	2 (4.0%)	0 (0.00%)	0 (0.00%)
Pancreas	All Platforms	15270	83 (0.54%)	306 (2.0%)	16 (0.10%)	14 (0.09%)	264 (1.7%)	13 (0.09%)	40 (0.26%)
Pancreas	FoundationOne CDx	8021	64 (0.80%)	135 (1.7%)	8 (0.10%)	11 (0.14%)	246 (3.1%)	4 (0.05%)	20 (0.25%)
Pancreas	FoundationOne Liquid CDx	3707	10 (0.27%)	72 (1.9%)	4 (0.11%)	2 (0.05%)	11 (0.30%)	7 (0.19%)	8 (0.22%)
Pancreas	GenMineTOP	340	0 (0.00%)	4 (1.2%)	1 (0.29%)	0 (0.00%)	1 (0.29%)	0 (0.00%)	2 (0.59%)
Pancreas	NCC Oncopanel	2450	6 (0.24%)	95 (3.9%)	3 (0.12%)	1 (0.04%)	3 (0.12%)	1 (0.04%)	8 (0.33%)
Pancreas	Guardant360	752	3 (0.40%)	0 (0.00%)	0 (0.00%)	0 (0.00%)	3 (0.40%)	1 (0.13%)	2 (0.27%)
Thoracic	All Platforms	6740	35 (0.52%)	1,488 (22.1%)	15 (0.22%)	94 (1.4%)	418 (6.2%)	134 (2.0%)	41 (0.61%)

Organ group	Platform	Cases	MSI-H	TMB-H	NTRK fusion/rearrangement	RET fusion/rearrangement	ERBB2 amplification	ALK fusion/rearrangement	BRAF V600E
Thoracic	FoundationOne CDx	4260	29 (0.68%)	1,021 (24.0%)	8 (0.19%)	64 (1.5%)	372 (8.7%)	83 (1.9%)	24 (0.56%)
Thoracic	FoundationOne Liquid CDx	1516	4 (0.26%)	276 (18.2%)	4 (0.26%)	18 (1.2%)	30 (2.0%)	33 (2.2%)	8 (0.53%)
Thoracic	GenMineTOP	207	0 (0.00%)	41 (19.8%)	1 (0.48%)	1 (0.48%)	6 (2.9%)	4 (1.9%)	1 (0.48%)
Thoracic	NCC Oncopanel	549	1 (0.18%)	150 (27.3%)	2 (0.36%)	10 (1.8%)	8 (1.5%)	9 (1.6%)	4 (0.73%)
Thoracic	Guardant360	208	1 (0.48%)	0 (0.00%)	0 (0.00%)	1 (0.48%)	2 (0.96%)	5 (2.4%)	4 (1.9%)
Genitourinary	All Platforms	8502	196 (2.3%)	792 (9.3%)	37 (0.44%)	11 (0.13%)	283 (3.3%)	18 (0.21%)	5 (0.06%)
Genitourinary	FoundationOne CDx	5125	150 (2.9%)	485 (9.5%)	19 (0.37%)	9 (0.18%)	263 (5.1%)	9 (0.18%)	2 (0.04%)
Genitourinary	FoundationOne Liquid CDx	2540	38 (1.5%)	220 (8.7%)	16 (0.63%)	2 (0.08%)	14 (0.55%)	8 (0.31%)	1 (0.04%)
Genitourinary	GenMineTOP	164	0 (0.00%)	20 (12.2%)	1 (0.61%)	0 (0.00%)	3 (1.8%)	1 (0.61%)	2 (1.2%)
Genitourinary	NCC Oncopanel	538	4 (0.74%)	67 (12.5%)	1 (0.19%)	0 (0.00%)	2 (0.37%)	0 (0.00%)	0 (0.00%)
Genitourinary	Guardant360	135	4 (3.0%)	0 (0.00%)	0 (0.00%)	0 (0.00%)	1 (0.74%)	0 (0.00%)	0 (0.00%)

For each row, the denominator for all biomarker columns is the number of cases shown in the Cases column.

NTRK and RET are operationalized as fusion/rearrangement classes, consistent with the manuscript-level operational definitions. ALK fusion/rearrangement and BRAF V600E are included only in the expanded practical-set context.

Supplementary Table S3. Distribution of the number of positive biomarkers per case overall and by organ group

Group	Primary 0 positive	Primary 1 positive	Primary 2 positive	Primary 3 positive	Primary 4+ positive	Expanded 0 positive	Expanded 1 positive	Expanded 2 positive	Expanded 3 positive	Expanded 4+ positive
Overall cohort	83,338 (85.6%)	12,037 (12.4%)	1,929 (2.0%)	39 (0.0%)	0 (0.0%)	81,432 (83.7%)	13,743 (14.1%)	2,057 (2.1%)	108 (0.1%)	3 (0.0%)
Biliary tract	7,709 (84.7%)	1,162 (12.8%)	229 (2.5%)	3 (0.0%)	0 (0.0%)	7,639 (83.9%)	1,223 (13.4%)	238 (2.6%)	3 (0.0%)	0 (0.0%)
Bowel	13,661 (86.5%)	1,888 (12.0%)	233 (1.5%)	9 (0.1%)	0 (0.0%)	12,971 (82.1%)	2,455 (15.5%)	296 (1.9%)	67 (0.4%)	2 (0.0%)
Breast	5,936 (79.2%)	1,404 (18.7%)	156 (2.1%)	2 (0.0%)	0 (0.0%)	5,912 (78.8%)	1,423 (19.0%)	160 (2.1%)	2 (0.0%)	1 (0.0%)
Esophagogastric	4,451 (76.4%)	1,152 (19.8%)	213 (3.7%)	7 (0.1%)	0 (0.0%)	4,439 (76.2%)	1,160 (19.9%)	217 (3.7%)	7 (0.1%)	0 (0.0%)
Gynecologic	9,117 (83.2%)	1,499 (13.7%)	344 (3.1%)	3 (0.0%)	0 (0.0%)	9,082 (82.8%)	1,533 (14.0%)	343 (3.1%)	5 (0.0%)	0 (0.0%)
Head and neck/thyroid	3,440 (85.4%)	541 (13.4%)	48 (1.2%)	1 (0.0%)	0 (0.0%)	2,971 (73.7%)	997 (24.7%)	61 (1.5%)	1 (0.0%)	0 (0.0%)

Group	Primary 0 positive	Primary 1 positive	Primary 2 positive	Primary 3 positive	Primary 4+ positive	Expanded 0 positive	Expanded 1 positive	Expanded 2 positive	Expanded 3 positive	Expanded 4+ positive
Liver	794 (88.5%)	90 (10.0%)	13 (1.4%)	0 (0.0%)	0 (0.0%)	790 (88.1%)	94 (10.5%)	13 (1.4%)	0 (0.0%)	0 (0.0%)
Central/peripheral nervous system	3,069 (91.4%)	227 (6.8%)	58 (1.7%)	3 (0.1%)	0 (0.0%)	2,898 (86.3%)	394 (11.7%)	62 (1.8%)	3 (0.1%)	0 (0.0%)
Other	8,211 (87.6%)	997 (10.6%)	155 (1.7%)	6 (0.1%)	0 (0.0%)	8,002 (85.4%)	1,191 (12.7%)	164 (1.8%)	12 (0.1%)	0 (0.0%)
Pancreas	14,654 (96.0%)	550 (3.6%)	65 (0.4%)	1 (0.0%)	0 (0.0%)	14,606 (95.7%)	595 (3.9%)	66 (0.4%)	3 (0.0%)	0 (0.0%)
Thoracic	4,872 (72.3%)	1,689 (25.1%)	176 (2.6%)	3 (0.0%)	0 (0.0%)	4,718 (70.0%)	1,823 (27.0%)	195 (2.9%)	4 (0.1%)	0 (0.0%)
Genitourinary	7,424 (87.3%)	838 (9.9%)	239 (2.8%)	1 (0.0%)	0 (0.0%)	7,404 (87.1%)	855 (10.1%)	242 (2.8%)	1 (0.0%)	0 (0.0%)

Counts summarize the number of positive biomarkers per case within the aggregated nationwide framework. Because exact pairwise or exact-combination overlap matrices were not available in the cumulative outputs, the overlap summary is limited to the number of positive biomarker classes per case.

Primary columns use the primary strict approved-set biomarker definition; expanded columns use the expanded practical-set definition.

Supplementary Table S4. Operational definitions of biomarker domains and case-level union endpoints

Biomarker/domain	Endpoint set	Operational definition used in this manuscript	Source field or event class available from the manuscript	Key interpretation caveat	Case-level exclusivity / overlap
MSI-H	Strict approved set	Counted when MSI-Status was reported as high or equivocal.	MSI-Status field in the aggregated outputs.	This is an operational definition for aggregated outputs. Including equivocal MSI status may overestimate MSI positivity if equivocal results are not confirmed.	Not mutually exclusive. A case can also be positive for another biomarker domain and is counted once in the union endpoint.
TMB-H	Strict approved set	Counted when TMB-Status was reported as high, or when TMB-Status was blank and the reported TMB value was ≥10 mutations/Mb.	TMB-Status and TMB value fields in the aggregated outputs.	The threshold reflects the manuscript-level operational rule and should not be interpreted as evidence of uniform cross-platform TMB calibration or identical TMB algorithms.	Not mutually exclusive. A case can also be positive for another biomarker domain and is counted once in the union endpoint.
NTRK fusion/rearrangement	Strict approved set	Restricted to rearrangement-class events annotated as fusion in NTRK1, NTRK2, or NTRK3.	Rearrangement-class output category; marker/gene token NTRK1, NTRK2, or NTRK3; change annotated as fusion.	This rule harmonizes aggregated output categories and does not establish a cross-platform biological truth state or equivalent fusion-detection sensitivity.	Not mutually exclusive. Rare co-occurrence with other domains is possible and handled through case-level union deduplication.
RET fusion/rearrangement	Strict approved set	Restricted to RET rearrangement-class events annotated as fusion/rearrangement events.	Rearrangement-class output category; marker/gene token RET; change annotated as fusion or rearrangement.	Fusion/rearrangement ascertainment may depend on specimen type, assay architecture, analytic pipeline, and reporting rules.	Not mutually exclusive. Rare co-occurrence with other domains is possible and handled through case-level union deduplication.
ERBB2 amplification	Strict approved set	Counted when ERBB2 copy-number amplification was reported.	Copy-number alteration/amplification event class; marker ERBB2.	Observed ERBB2 amplification frequency reflects surfaced output in routine CGP-tested	Not mutually exclusive. A case can also be TMB-H, MSI-H, or fusion/rearrangement-positive.

Biomarker/domain	Endpoint set	Operational definition used in this manuscript	Source field or event class available from the manuscript	Key interpretation caveat	Case-level exclusivity / overlap
				cases and is not a prevalence estimate in an untested population.	
ALK fusion/rearrangement	Expanded practical set only	Counted when ALK fusion/rearrangement events were reported.	Rearrangement-class output category; marker/gene token ALK.	Included as adjacent actionable context outside the primary strict approved-set endpoint; not part of the headline strict approved set.	Not mutually exclusive and contributes only to the expanded practical-set union endpoint.
BRAF V600E	Expanded practical set only	Counted when BRAF V600E was reported.	Sequence-variant/SNV event class; marker BRAF; change V600E.	Included as adjacent actionable context outside the primary strict approved-set endpoint; not part of the headline strict approved set.	Not mutually exclusive and contributes only to the expanded practical-set union endpoint.
Primary strict approved-set endpoint	Strict approved set	Case-level union of MSI-H, TMB-H, NTRK fusion/rearrangement, RET fusion/rearrangement, and ERBB2 amplification.	Derived case-level union across the five strict approved-set domains in the aggregated outputs.	Designed to avoid double counting across biomarker domains; it is not a direct estimate of biological prevalence or true patient-level therapeutic opportunity.	Each case is counted once if any strict approved-set biomarker domain is positive.
Expanded practical-set endpoint	Expanded practical set	Case-level union of the primary strict approved-set domains plus ALK fusion/rearrangement and BRAF V600E.	Derived case-level union across the expanded domain list in the aggregated outputs.	Secondary practice-facing context only; should not be conflated with the primary strict approved-set endpoint.	Each case is counted once if any expanded practical-set biomarker domain is positive.

The table summarizes manuscript-level operational definitions used for descriptive benchmarking in aggregated C-CAT outputs. These definitions are not intended to imply identical analytic sensitivity, equivalent assay coverage, or a harmonized biological truth state across platforms.

Supplementary Table S5. Platform and assay-context considerations for interpreting biomarker assessment across routine CGP platforms

Platform category	Specimen/testing context supported by the manuscript	Role in this manuscript	Biomarker assessment context	Important caveat for interpretation
FoundationOne CDx	Tissue-based CGP category in pooled specimen-context summaries.	Platform-specific descriptive category in routine CGP framework.	Observed outputs were summarized through the manuscript-level MSI/TMB/rearrangement-CNA-variant operational framework where available in the aggregated outputs.	Do not assume equivalent analytical sensitivity, coverage, or reporting behavior across all biomarker domains. Observed differences may reflect specimen availability, tumor content, platform architecture, reporting rules, organ mix, clinical pathway, and non-random platform selection.
FoundationOne Liquid CDx	Liquid-based CGP category in pooled specimen-context summaries.	Platform-specific descriptive category in routine CGP framework.	Observed outputs were summarized through the manuscript-level MSI/TMB/rearrangement-CNA-variant operational framework where available in the aggregated outputs.	Liquid-based results may be influenced by circulating tumor DNA shedding, tumor fraction, analyte structure, specimen timing, and non-random clinical selection. Do not interpret as assay superiority or inferiority.
GenMineTOP	Tissue-based CGP category in pooled specimen-context summaries.	Platform-specific descriptive category in routine CGP framework.	Observed outputs were summarized through the manuscript-level MSI/TMB/rearrangement-CNA-variant operational framework where available in the aggregated outputs.	Observed frequencies are surfaced routine-CGP signals and may be affected by panel architecture, analyte requirements, variant reporting rules, specimen quality, and organ-case composition.
NCC Oncopanel	Tissue-based CGP category in pooled specimen-context summaries.	Platform-specific descriptive category in routine CGP framework.	Observed outputs were summarized through the manuscript-level MSI/TMB/rearrangement-CNA-variant	Observed differences should be read as descriptive implementation patterns, not diagnostic-accuracy

Platform category	Specimen/testing context supported by the manuscript	Role in this manuscript	Biomarker assessment context	Important caveat for interpretation
			operational framework where available in the aggregated outputs.	comparisons, because patient-level platform selection and paired-assay data were unavailable.
Guardant360	Liquid-based CGP category in pooled specimen-context summaries.	Platform-specific descriptive category in routine CGP framework.	Observed outputs were summarized through the manuscript-level MSI/TMB/rearrangement-CNA-variant operational framework where available in the aggregated outputs.	Liquid-based testing may under-surface or differentially surface some domains depending on ctDNA shedding, tumor fraction, variant class, and reporting rules. Do not infer cross-platform sensitivity ranking.
Pooled tissue-based context	FoundationOne CDx, GenMineTOP, and NCC Oncopanel.	Coarse testing-context summary only.	Used to contextualize descriptive patterns across tissue-based CGP categories.	Not an exposure group and not a formal contrast with pooled liquid-based testing; organ mix and selection pathways are not randomized.
Pooled liquid-based context	FoundationOne Liquid CDx and Guardant360.	Coarse testing-context summary only.	Used to contextualize descriptive patterns across liquid-based CGP categories.	Not an exposure group and not a formal contrast with pooled tissue-based testing; organ mix and selection pathways are not randomized.

This table intentionally avoids unsupported claims about platform superiority, inferiority, or equivalent analytical sensitivity. Exact platform-specific coverage, TMB algorithms, and fusion-detection methodologies were not evaluated in this aggregated analysis and should be interpreted from authoritative platform documentation if needed.
