# Supplementary figures and images for: Implementation Benchmark of Tumor-Agnostic Eligibility Signals Across Routine Comprehensive Genomic Profiling Platforms in Japan: A Nationwide C-CAT Analysis
Source: Curr Oncol. 2026 May 30;33(6):324. doi: 10.3390/curroncol33060324 (PMC13297875; doi:10.3390/curroncol33060324)

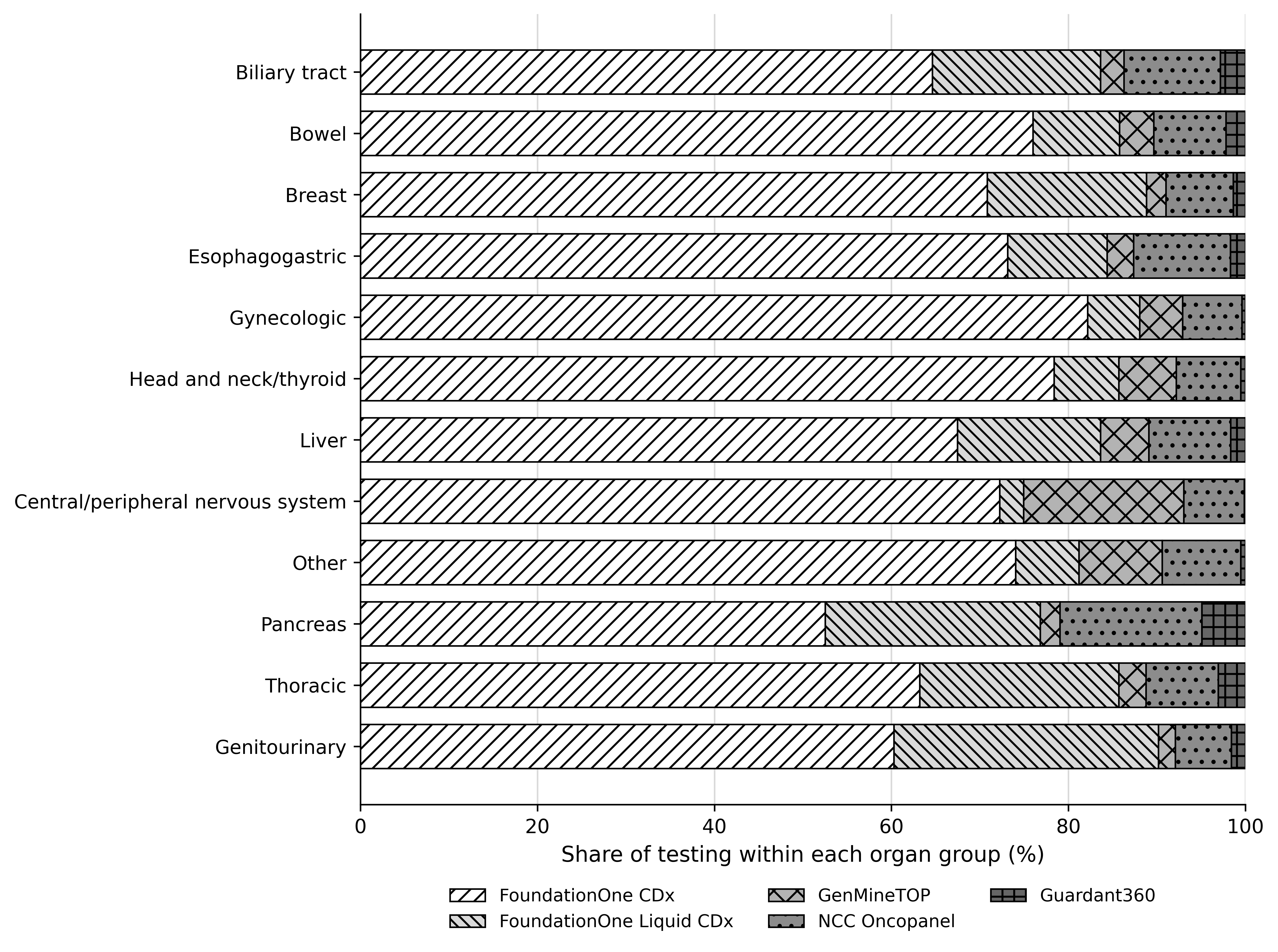

Supplement: Supplementary file 1 [file curroncol-33-00324-s001.zip › 33_Supplementary_FigureS2_Platform_Use_Distribution.tiff]
